# Associations between different measures of SARS-CoV-2 infection status and subsequent economic inactivity: A pooled analysis of five longitudinal surveys linked to healthcare records

**DOI:** 10.1371/journal.pone.0321201

**Published:** 2025-04-09

**Authors:** Richard J. Shaw, Olivia K. L. Hamilton, Rebecca Rhead, Richard J. Silverwood, Jacques Wels, Jingmin Zhu, Giorgio Di Gessa, Ruth C. E. Bowyer, Bettina Moltrecht, Michael J. Green, Evangelia Demou, Serena Pattaro, Paola Zaninotto, Andy Boyd, Felix Greaves, Nishi Chaturvedi, George B. Ploubidis, Srinivasa Vittal Katikireddi

**Affiliations:** 1 MRC/CSO Social and Public Health Sciences Unit, University of Glasgow, Glasgow, United Kingdom; 2 Centre for Longitudinal Studies (CLS), UCL Social Research Institute, University College London, London, United Kingdom; 3 Department of Psychological Medicine, King’s College London, London, United Kingdom; 4 MRC Unit for Lifelong Health and Ageing, University College London, London, United Kingdom; 5 Centre Metices, Université libre de Bruxelles, Brussels, Belgium; 6 Department of Epidemiology & Public Health, University College London, London, United Kingdom; 7 Department of Twin Research and Genetic Epidemiology, School of Life Course & Population Sciences, King’s College London, London, United Kingdom; 8 AI For Science & Government, Alan Turing Institute, London, United Kingdom; 9 Division of Women’s Community and Population Health, Department of Obstetrics and Gynecology, Duke University School of Medicine, Durham, NC, United States of America; 10 Scottish Centre for Administrative Data Research (SCADR), University of Glasgow, Glasgow, United Kingdom; 11 Population Health Sciences, University of Bristol, Bristol, United Kingdom; 12 Department of Primary Care and Public Health, Imperial College London, London, United Kingdom; World Health Organization, EGYPT

## Abstract

**Introduction:**

Following the acute phase of the COVID-19 pandemic, a record number of people became economically inactive in the UK. We investigated the association between coronavirus infection and subsequent economic inactivity among people employed pre-pandemic, and whether this association varied between self-report versus healthcare recorded infection status.

**Methods:**

We pooled data from five longitudinal studies (1970 British Cohort Study, English Longitudinal Study of Ageing, 1958 National Child Development Study, Next Steps, and Understanding Society), in two databases: the UK Longitudinal Linkage Collaboration (UKLLC), which links study data to NHS England records, and the UK Data Service (UKDS), which does not. The study population were aged 25-65 years between April 2020 to March 2021. The outcome was economic inactivity measured at the time of the last survey (November 2020 to March 2021). The exposures were COVID-19 status, indicated by a positive SARS-CoV-2 test in NHS records (UKLLC sample only), or by self-reported measures of coronavirus infection (both samples). Logistic regression models estimated odds ratios (ORs) adjusting for potential confounders including sociodemographic variables and pre-pandemic health.

**Results:**

Within the UKLLC sample (N = 8,174), both a positive SARS-CoV-2 test in NHS records (5.9% of the sample; OR 1.08, 95%CI 0.68-1.73) and self-reported positive tests (6.5% of the sample; OR 1.07, 95%CI 0.68-1.69), were marginally and non-significantly associated with economic inactivity (5.3% of the sample) in adjusted analyses. Within the larger UKDS sample (n = 13,881) reliant on self-reported ascertainment of infection (6.4% of the sample), the coefficient indicated a null relationship (OR 0.98, 95%CI 0.68-1.40) with economic inactivity (5.0% of sample).

**Conclusions:**

Among people employed pre-pandemic, testing positive for SARS-CoV-2 was not associated with increased economic inactivity, although we could not exclude small effects. Ascertaining infection through healthcare records or self-report made little difference to results. However, processes related to record linkage may introduce small biases.

## Introduction

Considerable economic disruption occurred internationally following the start of the COVID-19 pandemic [1], with many people leaving or losing their jobs [2]. In the USA this led to a drop in the labour force participation rate of nearly 1 percentage point, with the greatest drop in average yearly hours worked (around 23 hours) being for men with more than a high school education [2]. In the UK, coinciding with the spread of COVID-19, the number of economically inactive people increased dramatically, with 500,000 additional people being economically inactive in 2022 [3]. Driven principally by people over the age of 50, the number of older workers on unemployment-related benefits nearly doubled as a result of the pandemic, with the claimant count among the over 50s increasing by 280,000 between February 2020 and June 2020 [4]. This represents the largest increase in economic inactivity since records began in 1971 [5]. At the macro-level, UK labour market shortages have increased. At the individual-level, those affected face devastating financial and personal consequences [6].

Negative health consequences of severe acute respiratory syndrome coronavirus-2 (SARS-CoV-2) infection may have contributed to increased numbers of people leaving the workforce. Post-COVID-19 condition (also known as ‘long COVID’) has been linked to reductions in working capacity [7], substantial absences from work [8–10], and disrupted finances [11]. However, the studies in this area are generally limited, with most being based on small samples of people who have been hospitalised for COVID-19 [12], treated by primary care services [13], or based on cross-sectional surveys of people who were recruited because they reported having had COVID-19 [8, 9]. In addition, post-COVID-19 condition is partly defined on the basis that symptoms impact aspects of everyday functioning including work [14]. For some people, therefore, post-COVID-19 condition could be partly a consequence, as opposed to a cause, of their employment status [5].

Longitudinal population surveys (LPS) are an important source not only of economic activity data following the pandemic, but also socioeconomic conditions and health status prior to the pandemic, allowing for better adjustment of confounding. However, neither economic inactivity nor SARS-CoV-2 infection are typically common enough for analyses to be conducted using data from a single longitudinal study. A solution to these challenges is to pool data from multiple surveys and link them to National Health Service (NHS) data to provide standardised measures for SARS-CoV-2 positivity across studies. The UK Longitudinal Linkage Collaboration (UK LLC) Trusted Research Environment (TRE), which has linked NHS data to many major UK longitudinal surveys [15], makes pooled analysis feasible.

Using samples of LPS respondents who reported being employed or self-employed just before the pandemic, we aim to understand the relationship between two measures of COVID-19 status (a positive test for SARS-CoV-2 and a self-reported infection measure) and economic inactivity. When used to indicate COVID-19 status, both NHS records for SARS-CoV-2 tests and self-report of tests should provide similar results. Recall and specificity of test results have been shown to be high [16]. However, self-reported COVID-19 symptoms and test results have been demonstrated to show different relationships with symptoms [17] and this may be the case with employment related outcomes.

Firstly, we test associations between measures of COVID-19 status and economic activity in a sample from the UK LLC, which includes both COVID-19 status measures. However, as linkage consent limits the availability of data for some LPSs in the UK LLC, it is possible that this sample is subject to bias [18]. Secondly, therefore, we aimed to evaluate the consequences of potential consent bias by repeating analyses of the self-reported COVID-19 status measures using a larger sample from UK Data Service (UKDS).

## Methods

We use self-reported sociodemographic, economic and COVID-19 data on individuals from five UK LPSs, which are described in Table 1 and Appendix A in the S1 File: three age-homogenous cohort studies (the 1970 British Cohort Study; BCS70 [19], the 1958 National Child Development Study; NCDS [20] and Next Steps; NS [21]), and two population-based studies (Understanding Society; USoc [22, 23] and the English Longitudinal Study of Ageing; ELSA [24]). Each of these datasets included demographic, health and COVID-19 related variables, and information on employment status just before the pandemic, and employment status and economic activity during the pandemic. We pooled data across the five LPSs and derived two analytic samples, one within the UK LLC and the other within the UKDS:

**Table 1:** Showing the Study design and pre-pandemic and COVID-19 survey dates for each of the longitudinal population studies.

Longitudinal population study	Study design	Pre-pandemic survey date	Details of COVID-19 surveys (response rate)
1970 British Cohort Study (BCS70)	Children born in the UK in one week in 1970, with regular follow-up surveys from birth	July 2016 to July 2018 (Wave 10) & May 2020 (retro-recalled data)	Three surveys: May 2020 (40.4%), September-October 2020 (43.9%), February-March 2021 (40.0%)
National Child Development Study (NCDS)	Children born in the UK in one week in 1958, with regular follow-up surveys from birth	September 2013 – March 2014 (Wave 9) & May 2020 (retro-recalled data)	Three surveys: May 2020 (57.9%), September-October 2020 (53.9%), February-March 2021 (52.0%)
Next Steps (NS)	Sample recruited via secondary schools in England at around 13 years old, with regular follow-up surveys thereafter	August 2015 – September 2016 (Wave 8) & May 2020 (retro-recalled data)	Three surveys May 2020 (20.3%), September-October 2020 (31.8%), February-March 2021 (29.0%)
Understanding Society (USoc)	Nationally representative longitudinal household panel survey, based on a clustered-stratified probability sample of UK households, with participants surveyed annually	January 2015 –February 2020 (Participants most recent data before March 2020 from waves K to G)	Eight surveys: April (42.0%), May (35.1%), June (33.5%), July (32.6%), September (30.6%), November 2020 (28.6%), January (28.5%), March 2021 (30.2%)
English Longitudinal Study of Ageing (ELSA)	Nationally representative population study of individuals aged 50 + years living in private households in England, with biennial surveys and periodic refreshing of the sample to maintain representativeness	June 2018 – July 2019 (Wave 9)	Two surveys: June-July 2020 (75%), November-December 2020 (73%)

(1) Within the UK LLC TRE we selected people in England who consented to linkage to NHS data, were in employment just before the pandemic (see Appendix B in S1 File for definition), aged 25 years and over in the first COVID-19 survey they participated in, and aged 65 or under at the last COVID-19 survey they took part in (“UKLLC sample”; see Table 1 for further information on survey timings). The younger age limit was selected to exclude those of younger ages when education would be a common alternative to employment. The older limit reflects statutory retirement age. The UK LLC links data to NHS health records, so contains both the test-confirmed SARS-CoV-2 and the self-reported COVID-19 status variables in a sample restricted to those who have consented to record linkage. NHS records include positive tests for SARS-CoV-2 from the English NHS Digital COVID-19 Second Generation Surveillance (COVIDSGSS), NHS Digital Npex, and NHS Digital Covid-19 UK Non-hospital Antibody Testing Results [15].
(2) The UKDS sample differs from the UKLLC sample in three main ways: 1) data are not linked to NHS records, therefore, the sample is larger as it is not limited by consent to linkage; 2) because the data are not linked, they do not include SARS-CoV-2 test records, only the self-reported COVID-19 measure; 3) the UKDS sample includes people from all four UK nations, not just England. For our primary analyses, we derived a sample by applying the same age restrictions as above to residents of all four UK nations. To aid comparability with the UKLLC sample, we then repeated analyses in the UKDS, restricting the sample to residents of England only.

### Variables

#### Outcomes.

The primary outcome is economic inactivity, which categorises participants into the reference category of economically active (i.e., those who are in paid employment, or seeking paid employment), or economically inactive (i.e., those who are neither in paid employment, nor looking for work). Further sensitivity analyses were conducted, using employment status as the outcome, categorised as those in paid employment (reference category) versus those not in employment. The distinction between the two measures is that being economically active includes those who are in paid employment and those who are unemployed but actively seeking work. In contrast, for the employment status measure, the non-employed category includes not only economically inactive people, e.g., retired or long-term sick but also unemployed people who are looking for work and who would be classified as economically active. To aid consistency, outcome data are taken from a single wave for approximately the same period of each longitudinal cohort survey, which ranged from November/December 2020 to March 2021 (details are given in Table 1). More extensive details on how the outcome variables were defined are provided in Appendix C in S1 File.

#### Exposures.

Two measures of COVID-19 status were used:

(1) An NHS record indicating that participants tested positive for SARS-CoV-2 prior to the survey during which economic inactivity or employment status was ascertained (UKLLC sample only).
(2) A self-reported COVID-19 status variable, whereby participants were categorised as not having COVID-19, having suspected COVID-19, or having test-confirmed COVID-19, according to self-reported measures up to and including the survey used to record the outcome (see Appendix D in S1 File; both UKLLC and UKDS samples).

### Potential confounding variables

We had available the following demographic variables: age at time of outcome (standardised linear and quadratic terms); sex (male versus female); household composition at start of pandemic (alone, partner no children, partner and children/grandchildren, no partner and children/grandchildren, other (e.g., living with housemate(s)); self-reported key worker status during the pandemic (no/yes); and ethnicity (White, Asian, Black, Mixed, Other). NHS records, if available, were used if ethnicity data were missing (5.4% of the UKLLC sample). For the UKDS analyses ethnicity was not used. BCS70 ethnicity data was restricted to participants of a sweep that occurred in the year 2000, and only a binary ethnicity measure was available for ELSA. Adjusting for ethnicity would have led to a smaller sample size for the addition of a variable of limited utility. The pre-pandemic socioeconomic confounders were the National Statistics Socio-Economic Classification (NS-SEC: higher management and professional, lower management and professional, intermediate, small employer, lower supervisory and technical, semi-routine, routine, unclassifiable) and the highest level of education (harmonised using National Vocational Qualifications (NVQ) or academic equivalents see Schneider 2011 [25] into NVQ4 or 5, NVQ3, NVQ2 or 1, none, unclassifiable). To adjust for health, we used pre-pandemic self-rated health (excellent, very good, good, fair or poor) and whether a person reported having been advised to ‘shield’ (i.e., self-isolate; no versus yes) during the pandemic, based on whether or not they reported receiving a letter from the NHS indicating that they were at risk of severe illness if they caught coronavirus. NS-SEC and self-rated health data for ELSA and USoc were collected prior to the pandemic, whereas retrospective measures collected during the first pandemic sweep were used for NCDS, BCS70 and NS.

### Statistical analyses

We carried out logistic regression models to test the following associations:

In the UK LLC

**Model 1:** NHS test records for SARS-CoV2 ~  economic activity +  potential confounding variables
**Sensitivity analysis 1:** NHS test records for SARS-CoV2 ~  employment status +  potential confounding variables..
**Model 2:** Self-reported COVID-19 status ~  economic activity +  potential confounding variables
**Sensitivity analysis 2**: Self-reported COVID-19 status ~  employment status +  potential confounding variables

In the UKDS:

**Model 3:** Self-reported COVID-19 status ~  economic activity +  potential confounding variables
**Sensitivity analysis 3:** Self-reported COVID-19 status ~  employment status +  potential confounding variables
**Sensitivity analysis 4 (England-only sample):** Self-reported COVID-19 status ~  economic activity +  potential confounding variables.
**Sensitivity analysis 5 (England only sample):** Self-reported COVID-19 status ~  employment status +  potential confounding variables

In addition, we carry out analyses stratifying the full UKDS sample by the following characteristics: age (under 50 years versus 50 and over), sex and NS-SEC (higher management, administrators, and professionals versus intermediate, service and routine), and self-rated health (excellent, very good, good versus fair or poor). We formally tested for effect modification by these characteristics using likelihood ratio tests.

We used complete case analysis to address missing data. Regression analyses tend to be robust to missing data and more appropriate than other alternatives such as weighting and multiple imputation which could amplify biases when the data is missing not at random [26, 27].

The code to derive all the variables and carry out the analyses is available from the UK LLC Github repository (https://github.com/UKLLC/llc_0010). The analyses and coding of the survey data were carried out using Stata 17.0, while the graphs and coding of NHS data were carried out using R 4.3.1

### Ethics statement

The analyses use secondary data obtained from the UK LLC and the UKDS, ethical clearance was obtained by the UK LLC and the studies held within it. The UK LLC is a Trusted Research Environment developed and operated by the Universities of Bristol and Edinburgh using an underlying ‘Secure eResearch Platform’ infrastructure (https://serp.ac.uk/) provided by Swansea University for longitudinal research. The UK LLC TRE is designed to host de-identified data from many interdisciplinary, longitudinal population studies; to systematically link these to participants’ health, administrative and environmental records; and to provide a secure analysis environment. This project has been approved by the UK LLC and its contributing data owners and information on this project and its outputs can be accessed via UK LLC’s website (Data Use Register | UK Longitudinal Linkage Collaboration (ukllc.ac.uk)) and UK LLC’s GitHub (UK Longitudinal Linkage Collaboration GitHub). The UK LLC has ethical approval from the Health Research Authority Research Ethics Committee (Haydock Committee; ref: 20/NW/0446). More information on ethical approval for each longitudinal population study is available in the Appendix A in S1 File.

## Results

**Fig 1:**
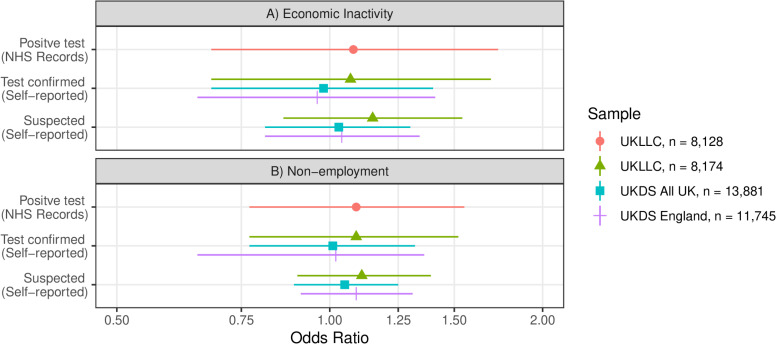
Odds ratios for economic inactivity and non-employment by COVID-19 status in four subsamples of five UK Longitudinal Population Studies. Analyses were adjusted for age, sex, household composition, ethnicity (UKLLC only), longitudinal study, country (UKDS only), shielding status, self-rated health, NS-SEC, keyworker status and education.

The UKLLC sample included 8,174 people with complete data (full details on the selection process are given in Fig 1 in S1 File), of whom 479 people (5.9%) had an NHS record of a positive test for SARS-CoV-2, 528 (6.5%) self-reported having had COVID-19 confirmed by a test, and 1,164 (14.3%) suspected they had had COVID-19 (Table 2).

**Table 2:** Characteristics of participants in the UKLLC and UKDS analytic samples.

Variable	UKLLC		UKDS		Variable	UKLLC		UKDS	
	n	%	n	%		n	%	n	%
**Outcome**					**Potential confounders cont…**				
*Economic activity*					*Age categories*				
Active	7,718	94.4	13,141	94.7	25 to 29 years	156	1.9	349	2.5
Inactive	429	5.3	689	5.0	30 to 39 years	1,385	16.9	2,362	17.0
Missing	27	0.3	51	0.4	40 to 49 years	792	9.7	1,623	11.7
					50 to 59 years	3,378	41.3	5,757	41.5
*Employment status*					60 to 65 years	2,463	30.1	3,790	27.3
Employed	7,296	89.3	12,433	89.6					
Non-employed	847	10.4	1,380	9.9	*Sex*				
Missing	31	0.4	68	0.5	Male	3,543	43.3	5,917	42.66
					Female	4,631	56.7	7,964	57.4
**Exposures**									
*Positive test in NHS COVID-19 records*					*Household composition*				
No	7,695	94.1	NA	NA	Alone	1,198	14.7	2,039	14.7
Yes	479	5.9	NA	NA	Partner	2,722	33.3	4,441	32.0
					Partner & children	3,347	41.0	5,838	42.1
Self-reported COVID-19					Children without partner	493	6.0	874	6.3
No COVID-19	6,436	78.7	11,082	79.8	Other person	414	5.1	689	5.0
Suspected	1,164	14.2	1,918	13.8					
Test confirmed	528	6.5	881	6.4	*Ethnicity*				
Missing	46	0.6	NA	NA	White	7,657	93.7	NA	NA
					Asian	258	3.2	NA	NA
**Potential confounders**					Black	105	1.3	NA	NA
*Shielding*					Mixed	106	1.3	NA	NA
No	7,811	95.6	13,325	96.0	Other	48	0.6	NA	NA
Yes	363	4.4	556	4.00					
					*Education*				
*Self-rated health*					NVQ 4 or 5	4,080	49.9	6,606	47.6
Excellent	1,099	13.5	1,875	13.5	NVQ 3	1,193	14.6	1,852	13.3
Very Good	3,350	41.0	5,677	40.9	NVQ 1 or 2	1,969	24.1	3,167	22.8
Good	2,701	33.0	4,565	32.9	None	596	7.3	1,135	8.2
Fair	894	10.9	1,538	11.1	Unclassifiable	336	4.1	1,121	8.1
Poor	130	1.6	226	1.6					
					*Longitudinal sample*				
*NS-SEC*					BCS70	1,832	22.1	2,960	21.1
Higher manager/professional	1,472	18.0	2,452	17.7	ELSA	815	9.9	949	6.8
Lower manager/professional	2,568	31.4	4,411	31.8	NCDS	1,636	19.8	2,468	17.8
Intermediate	1,326	16.2	2,262	16.3	Next Steps	907	11.0	1,328	9.6
Small employer	541	6.6	940	6.8	Understanding Society	3,086	37.3	6,176	44.5
Lower supervisory/technical	377	4.6	678	4.9					
Semi-routine	952	11.7	1,716	12.4	*Country*				
Routine	478	5.9	819	5.9	England	8,276	100	11,745	84.6
Unclassifiable	460	5.6	603	4.3	Scotland			1,017	7.3
					Wales			596	4.3
*Keyworker status*					Northern Ireland			290	2.1
No	4,558	55.8	7,742	55.2	Elsewhere			233	1.7
Yes	3,616	44.2	6,297	44.9					

The full UKDS sample included 13,881 people (see S2 Fig in S1 File). Proportions of participants reporting COVID-19 confirmed by a test or suspected COVID-19 were slightly lower than the UKLLC sample (6.4% and 13.8% respectively).

The percentage of people reporting that they were economically inactive was similar in the UKLLC and UKDS samples (UKLLC: 5.3%, n = 429; UKDS: 5.0%, n =  689), as was the case for people reporting that they were unemployed (UKLLC: 10.4%, n =  847; UKDS: 9.9%, n = 1,380). The majority of the UKLLC and UKDS samples were over the age of 50 (UKLLC: 71.5%, n =  5,841; UKDS: 68.8%, n =  9,547). Both samples also over-represented females (UKLLC: 56.7%, n =  4,6315; UKDS: 57.4%, n =  7,964).

### Associations between NHS-record of SARS-CoV-2 and economic inactivity

For the UKLLC sample, results of adjusted analyses indicated that having an NHS record of a positive test for SARS-CoV2 resulted in a weak non-significant increased risk of economic inactivity (OR 1.08, 95% CI 0.68 to 1.73; see panel A, Fig 1 and Table 1 in S1 File).

### Associations between self-reported COVID-19 status and economic inactivity

In the UKLLC sample, relative to those who did not report having COVID-19, those who self-reported having had test confirmed COVID-19, had a weak non-significant increased risk of economic inactivity (OR 1.07, 95% CI 0.68 to 1.69), which was very close to the associations indicated by NHS test records. In the full UKDS sample, associations between self-reported test-confirmed COVID-19 and economic activity were closer to 1 (OR 0.98, 95% CI 0.68 to 1.40, see Table 1 in S1 File). Results were similar in the UKDS England-only sensitivity analyses (OR 0.96, 95% CI 0.65 to 1.41).

In the UKLLC sample, self-reported suspected COVID-19 was associated with a possible albeit non-significant increased risk of economic inactivity (OR 1.15, 95% CI 0.86 to 1.54). Estimates for the whole UKDS sample (OR 1.03, 95% 0.81 to CI 1.30; see Table 1 in S1 File) and the UKDS England (OR 1.04 95% 0.81 to 1.34) sample were much closer to 1.

### Sensitivity analyses: associations between measures of COVID-19 status and non-employment

Associations between different measures of COVID-19 status and non-employment were comparable to the above described associations with economic inactivity, in the UKLLC and both full and England-only UKDS samples (see panel b of Fig 1).

### Stratified analyses

The relationship between COVID-19 status and the economic inactivity stratified by age, sex, NS-SEC and self-reported health are shown in Table 3. While there are modest variations between the strata, neither the stratified results nor formal testing supported the idea that the relationship between COVID-19 and economic inactivity varied by any of these factors.

**Table 3:** Adjusted^1^ associations between self-reported COVID-19 status and economic stratified by age, sex, NS-SEC, self-reported health and keyworker status using the UKDS full sample.

COVID-19 status	Economic Inactivity			
Ref = No Covid	Odds Ratio	95% CI		p
**Age**				
*People under 50*				
Suspected	0.93	0.53	1.63	0.794
Test confirmed	1.35	0.68	2.67	0.393
*People 50 and over*				
Suspected	1.03	0.79	1.34	0.835
Test confirmed	0.92	0.60	1.40	0.699
**Sex**				
*Male*				
Suspected	1.06	0.71	1.56	0.789
Test confirmed	0.91	0.48	1.72	0.765
*Female*				
Suspected	1.01	0.74	1.36	0.971
Test confirmed	1.05	0.68	1.62	0.830
**NS-SEC**				
*Higher occupations*				
Suspected	1.28	0.92	1.79	0.144
Test confirmed	1.14	0.67	1.95	0.629
*Intermediate and routine occupations*				
Suspected	0.80	0.55	1.17	0.250
Test confirmed	0.97	0.60	1.62	0.940
**Self-rated health**				
*Excellent or good health*				
Suspected	1.10	0.84	1.42	0.492
Test confirmed	0.87	0.57	1.32	0.513
*Fair or poor health*				
Suspected	0.76	0.41	1.40	0.373
Test confirmed	1.67	0.81	3.44	0.167
**Key worker status**				
*Not keyworkers*				
Suspected	1.10	0.83	1.45	0.504
Test confirmed	1.16	0.78	1.85	0.409
*Key workers*				
Suspected	0.83	0.51	1.34	0.440
Confirmed	0.72	0.37	1.39	0.329

^1^Analyses were adjusted for age, sex, household composition, longitudinal study, country, shielding status, self-rated health, NS-SEC, keyworker status, and education (stratification variable was omitted).

## Discussion

Using data from five UK-based LPSs, our analyses aimed to understand the relationship between two measures of COVID-19 status (a positive test for SARS-CoV-2 and a self-reported infection measure) and economic inactivity. Results suggest that having SARs-CoV-2 confirmed by a test is either not associated or only confers a small increased risk of being economically inactive. Despite reasonably large sample sizes (approximately 8,000 in the UKLLC and 14,000 in the UKDS), the width of confidence intervals does not rule out stronger associations. There was little evidence to suggest that this varied by important characteristics such as age, sex, education or socio-economic classification.

Comparing samples, we find slightly higher risks of economic inactivity in the UKLLC sample as opposed to the UKDS sample. This small difference is unlikely to be the result of using different indicators for COVID-19 status. Within the UKLLC sample, the results using both the self-reported and NHS records are almost the same. Similarly, the differences between the UKLLC and UKDS samples are unlikely to be due to the geographic differences between the samples. There was little difference between the associations for the self-reported measure of COVID-19 between the UKDS sample restricted to England and the sample for the whole of the UK. One possible explanation is the requirement for consent to NHS linkage. For some of the studies included in the UKLLC, linkage to NHS records was based on consent. For most studies, including BCS70, NCDS, ELSA, and NS, consent for record linkage was collected prior to the COVID-19 pandemic. USoc local governance requirements demanded specific consent for inclusion in UK LLC, and this was acquired during the 8^th^ pandemic survey in March 2021. It is possible that differences between those who consented to linkage and those who did not may have introduced a small bias, which would be consistent with other studies [28]. Given that data in the UKLLC sample is only available for those who consented, it is not currently possible to model those biases directly. However, there are plans to obtain consent for more people. Given the current data limitations, we believe that the sensitivity analysis comparing the UKDA sample and UKLLC sample is a reasonable alternative.

For people employed prior to the pandemic, we found odds ratios for the association between testing positive for COVID-19 and economic activity that are close to one, albeit with wide confidence intervals. The current literature is focused on post-COVID condition (PCC). A non-peer reviewed initial rapid scoping review suggests that the socioeconomic consequences of PCC are considerable [12]. However, the review also indicated that long-term absences from work may only occur for 20% of people with PCC. A more recent study from the USA found only a modest increased risk of unemployment (OR 1.23 95% CI 1.02-1.48) among people with PCC compared to those who only had acute infection [29]. Another study from British Columbia [30] found strong evidence of PPC being related to any health-related adverse work outcome (OR 2.6 95% CI 1.7 to 4.0). However, more modest associations with a high degree of uncertainty were found for outcomes indicating a departure from the labour force, for example “retiring earlier than planned” (OR 1.6 95% 0.2 to 11.8) and “going on indefinite sick leave “(1.6 95% CI 0.5 to 5.0). PPC only arises in a small proportion of COVID-19 cases [31], and any possible impact on the workforce is potentially due to large numbers of people being infected with COVID-19 rather than a particular strong association [12]. It is therefore unsurprising that our study finds point estimates that are close to 1. It is possible that the consequences of COVID-19 may be greater for those on the periphery of the labour market, and an alternative approach would be to focus studies on high-risk groups for whom the outcome and/or the exposure might be more common. The principal initial drivers of the rise in economic inactivity appear to be age and poor health [3]. We included these as potential confounders and found associations consistent with this. Evaluating the economic impacts of COVID-19 is considered a priority [32]. Large-scale administrative data sets may be the only resource that provides sufficient precision to evaluate what may be small widespread effects. It should also be noted that the individual-level economic consequences of COVID-19 may be country specific due to differing labour markets and welfare systems.

### Strengths and limitations

A strength of our study is that we used two distinct measures of COVID-19 status: a directly observed positive test for SARS-CoV-2 derived from NHS digital data and a self-reported measure of test-confirmed COVID-19. Conceptually, test-confirmed SARS-CoV-2 is much more common than other potential measures related to COVID-19 such as post COVID-19 condition, which is experienced by only a small proportion of those who are infected [31]. In addition, test-confirmed SARS-CoV-2 is not dependent on criteria related to daily functioning which may include employment [14], which are likely to be the result of multiple causes not just COVID-19, and could introduce biases. However, the use of a test to ascertain COVID-19 means that poorer access to testing is a possible source of bias that could lead to an underestimation of effects [33]; we hope that adjusting for keyworker status minimised any impact of this. The use of test-confirmed SARS-CoV-2 will include asymptomatic, mild cases of COVID-19, and may exclude those unable or unwilling to be tested, thus would be expected to have weaker associations than measures of more severe COVID-19.

A further strength of our study is that the use of longitudinal survey data meant we could control for important pre-pandemic potential confounding factors such as education and occupational class. However, it is possible that we could not fully adjust for factors that might lead to behavioural changes that reduce the risk of exposure to the SARs-CoV-2 virus and labour market engagement. To mitigate against this, we did adjust for shielding, keyworker status, age and self-rated health. The UK LLC is planning to add linked administrative employment data which may extend the scope and period of employment outcomes that can be investigated.

Finally, pooling data across studies has enabled a much larger sample size than any of the individual studies alone would have allowed. Using the UK LLC TRE, pooling data has enabled the use of more detailed ethnicity information than is commonly available for BCS70, ELSA and NCDS, and a more detailed level of education and social class than employed in other approaches such as pooled meta-analysis, which have been used to combine longitudinal population studies in other papers [34].

One limitation of our work was that we did not have consistent Standard Occupational Classification (SOC) codes available across the cohorts. However, by adjusting for keyworker status we aimed to reduce any biases arising from increased exposure to COVID-19 among those in jobs which were more secure during this period. We only have information on COVID-19 status, economic inactivity and employment status covering the early pandemic periods, until March 2021. This was before most people had access to vaccines and was when COVID-19 posed the greatest health risk. However, it was also a period during which other policies and practices were in place such as furlough [35] and homeworking [34], and these may have enabled people to stay economically active. The economic consequences of SARS-CoV-2 infection for individuals may be different after March 2021, and the restricted period for which we have data also limited our ability to investigate longer term consequences.

For the included LPSs, the UKLLC sample only included people who consented to having their survey data linked to NHS records. In addition, despite being embedded within long standing cohorts, survey responses during the pandemic were lower than typically achieved. Thus, there may be some selection biases restricting generalisability of results. In line with this, we did find slightly higher point estimates for the associations between suspected COVID-19 and the outcomes in the UKLLC sample, than the more general population sample in the UKDS. Regression analyses tend to be robust to missing data and more appropriate than other alternatives such as weighting and multiple imputation, which could amplify biases when the data is missing not at random [26,27]. There is also the possibility that linkage errors and other errors with administrative data could potentially result in misclassification bias [18]. It should be noted that for the UKLLC sample, slightly more people (see Table 2 in S1 File) reported having had COVID-19 confirmed with a test in the self-reported data, than in the NHS test records.

### Interpretation and conclusions

Among people employed immediately prior to the COVID-19 pandemic, positive SARS-CoV-2 infection status was either weakly or not associated with increased economic inactivity. Whether the COVID-19 status measure was based on NHS records, or a self-report survey made little difference to results. It is possible that effect sizes could be biased by processes related to data linkage. Definitive answers may require investments in well-designed studies that evaluate population health on a scale that was inconceivable prior to the pandemic.

## Supporting information

S1 File The supplementary materials contain ethics and data access statements for each study. They also contain explanations of how pre-pandemic employment, economic activity, employment status, and self-reported COVID-19 were measured for each study. Also included are two tables of additional results, and two flow charts showing how the analytic samples were derived.
(DOCX)
